# A *De Novo* Missense *MYLK* Variant Leading to Nonsyndromic Thoracic Aortic Aneurysm and Dissection Identified by Segregation Analysis

**DOI:** 10.1155/2024/4281972

**Published:** 2024-08-16

**Authors:** Daigo Nishijo, Hiroki Yagi, Nana Akiyama, Norifumi Takeda, Masahiko Ando, Haruo Yamauchi, Norihiko Takeda, Issei Komuro

**Affiliations:** ^1^ Department of Cardiovascular Medicine Graduate School of Medicine The University of Tokyo, Tokyo, Japan; ^2^ Marfan Syndrome Center The University of Tokyo Hospital, Tokyo, Japan; ^3^ Department of Genomic Medicine The University of Tokyo Hospital, Tokyo, Japan; ^4^ Department of Cardiac Surgery The University of Tokyo Hospital, Tokyo, Japan; ^5^ Department of Frontier Cardiovascular Science Graduate School of Medicine The University of Tokyo, Tokyo, Japan; ^6^ International University of Health and Welfare, Tokyo, Japan

## Abstract

Nonsyndromic hereditary thoracic aortic aneurysm and dissection (TAAD) is an autosomal dominant disease; however, it is frequently difficult to identify the causative genes. We report in this study a 33-year-old Japanese male with TAAD (Stanford type A) that is complicated with severe aortic regurgitation. There was no family history of aortic diseases in the patient nor any specific clinical features suggestive of connective tissue diseases, such as Marfan syndrome. Genetic testing identified candidate causative variants in two different genes: *MYLK* (c.4819G > A, p.[Gly1607Ser]) and *FBN1* (c.365G > A, p.[Arg122His]). Familial cosegregation analysis revealed that the novel de novo *MYLK* variant was present only in the proband, and the *FBN1* variant was also found in his nonaffected mother, and thus the *MYLK* variant was classified as likely pathogenic. *MYLK* is a causative gene for nonsyndromic TAAD that requires careful management; however, the number of reports is limited. Accumulating data on the pathogenicity of rare variants by performing a comprehensive pedigree analysis would help establish better treatment strategies for life-threatening hereditary TAAD cases.

## 1. Introduction

Nonsyndromic hereditary thoracic aortic aneurysm and dissection (TAAD) is an autosomal dominant disorder with a wide range of clinical variabilities and incomplete penetrance [1]; however, its natural history of progress is still unknown. Most nonsyndromic TAAD cases are diagnosed due to early-onset age or family history, and the identification of pathogenic variants may provide prognostic and risk stratification information to the proband and their family members. Prophylactic aortic surgery is advised for the most severe type, which can be life-threatening, based on the specific genetic variant and aortic diameter [2–4].

Recently, the discovery of multiple genes associated with nonsyndromic TAAD was accelerated by next-generation sequencing technology [5–7], and this trend has made genetic testing indispensable for diagnosing genetic diseases and in their subsequent therapeutic management. Currently, rare variants and polymorphisms in *FBN1* (OMIM∗134797), which cause Marfan syndrome (MFS), have also been reported to be involved in nonsyndromic TAAD [8, 9]; however, when assessing the pathogenicity of unclassified variants, it is important to ensure that preventive measures are taken.

*MYLK* (OMIM∗600922) encodes myosin light chain kinase (MLCK), which is expressed in smooth muscle cells and phosphorylates the regulatory light chain, resulting in muscle contraction. Few research has been reported on missense variants, even though *MYLK* pathogenic variants have been found to account for 1% of nonsyndromic hereditary TAAD, with the majority likely being explained by frameshift and nonsense variants [10, 11]. In this study, we report a young nonsyndromic male with aortic dissection carrying two missense variants of unknown significance in *MYLK* and *FBN1*, whose segregation analysis helped evaluate their pathogenicity and was useful for genetic counseling in the proband and his families.

## 2. Materials and Methods

### 2.1. Ethical Compliance

The University of Tokyo Hospital's Institutional Ethics Committee approved the genetic testing for hereditary TAAD (Reference No. G-1538), and the patient gave written informed consent.

### 2.2. Genetic Analyses of TAAD

The Kazusa DNA Research Institute (Chiba, Japan) provided hybridization capture-based gene panel testing for hereditary TAAD under the coverage of Japanese health insurance. This panel testing contained the following genes: *FBN1, TGFBR1* (OMIM∗190181), *TGFBR2* (OMIM∗190182), *TGFB2* (OMIM∗190220), *TGFB3* (OMIM∗190230), *SMAD3* (OMIM∗603109), *ACTA2* (OMIM∗102620), *MYH11* (OMIM∗160745), *MYLK*, and *COL3A1* (OMIM∗120180) [12, 13]. The GenBank reference sequences and version numbers were as follows: FBN1 (NM_000138.4), TGFBR1 (NM_004612.3), TGFBR2 (NM_003242.5), TGFB2 (NM_003238.3), TGFB3 (NM_003239.4), SMAD3 (NM_005902.3), ACTA2 (NM_01613.4), MYH11 (NM_002474.2), MYLK (NM_053025.3), and COL3A1 (NM_00090.3).

### 2.3. Segregation Analysis

Segregation analysis of the candidate variant using Sanger sequencing was carried out in additional family members. For genetic testing for *MYLK* and *FBN1* variants, genomic DNA isolated from the white blood cells of the proband and his parents was used. In order to amplify exon 28 of *MYLK* and exon 5 of *FBN1*, intron-specific primers flanking exons were designed for polymerase chain reaction (PCR). PCR was carried out using the following primers located in exon 28 of *MYLK* (forward, 5′-GGGCTTTCCTGCCAATTTCTCCTACCCCC-3′, and reverse, 5′-GGGGCAGGATTCAAACCTGGGAAGTCTTC-3′) and exon 5 of *FBN1* (forward, 5′-GGAGTCAAGCAAGATGGAGC-3′, and reverse, 5′-AGCTGACACTACTTTTCCATTCT-3′). All sequencing procedures were performed using forward and reverse primers, as mentioned previously [14].

### 2.4. Histological Analysis of Human Aortic Tissue

The Institutional Ethics Committee of the University of Tokyo Hospital approved the examination of human aortic tissue (Reference No. 2233-7). Aortic root tissue samples were obtained from the patient (MT65) (III-1) who had elective aortic root surgery. After written informed consent was obtained, control aortic tissue samples were taken from a 43-year-old heart transplant recipient (MT21) who had dilated cardiomyopathy but no aortic aneurysm annulus ectasia (Valsalva 27 mm). The aortic tissue samples were fixed in 10% formalin, embedded in paraffin, and sectioned at a thickness of 5 *μ*m. Three serial sections were stained with Elastica van Gieson and Masson's trichrome, anti-phospho-Smad2 (Ser465/Ser467) antibody (Cell Signaling Technology, #3108, 1 : 200), and anti-phosphorylated ERK1/2 (Thr202/Tyr 204) antibody (Cell Signaling Technology, #9101, 1 : 200), using a VECTASTAIN ABC Kit (Vector Laboratories, PK-4001) and 3, 3′-diaminobenzidine tetrahydrochloride (Vector Laboratories, SK-4100).

## 3. Results

### 3.1. Case Presentation

**Figure 1:**
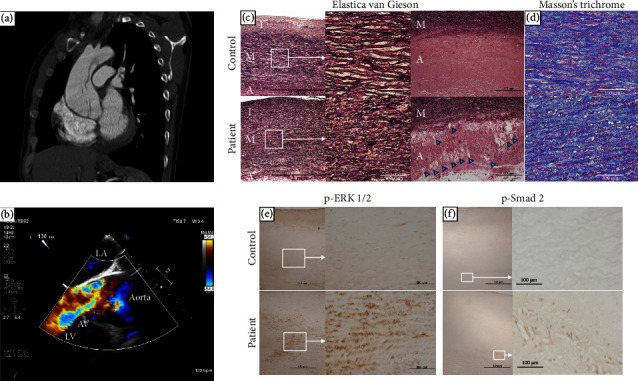
Japanese case of familial TAAD presenting with aortic disease. (a) Dissection from the aortic root to the aortic arch as shown on contrast-enhanced computed tomography (sagittal cross section). The maximum diameter of the ascending aorta was 55 mm and the sinus of Valsalva was 36 mm. (b) Transesophageal echocardiogram reveals a flap in the aortic root and severe aortic insufficiency. (c, d) Histological analysis with Elastica van Gieson stain and Masson's trichrome stain in the ascending aorta of the patient (MT65) and a control undergoing heart transplant (MT21). Abundant vasa vasorum in the patient's adventitia was observed (blue arrowheads). (e, f) Immunostaining for phosphorylated ERK1/2 (p-ERK1/2) and phosphorylated Smad2 (p-Smad2) in the ascending aorta of the patient (MT65) and a control undergoing heart transplant (MT21). TAAD, thoracic aortic aneurysm and dissection; LA, left atrium; AV, aortic valve; LV, left ventricle; I, intima; M, media; A, adventitia.

The patient was a 33-year-old Japanese male without apparent medical history. He suddenly developed chest pain and was transferred to a nearby hospital in May 2021. Computed tomography (CT) showed a 45 mm dilatation of the ascending aorta, but aortic dissection was not widely suspected. He kept experiencing symptoms of shortness of breath and chest pain on exertion afterward, and 3 months later, he returned to the outpatient clinic. Cardiac enlargement was observed on chest X-ray, and severe aortic insufficiency with ascending aorta enlargement was shown on echocardiogram. Contrast-enhanced CT revealed a Stanford type A false lumen aortic dissection that extended from the aortic root to the aortic arch without organ malperfusion. The maximum diameter of the ascending aorta increased to 55 mm compared to 3 months ago (Figure 1(a)). He was transferred to our university hospital, where he was immediately admitted for further evaluation, and transesophageal echocardiogram showed a false lumen at the aortic root and severe aortic insufficiency due to the enlargement of aortic valve ring (Figure 1(b)). Symptoms of heart failure were seen, and the diameter of the ascending aorta increased over the past few months; therefore, elective surgery was performed. Valve-sparing aortic root replacement (David procedure) and aortic valvuloplasty were performed. The thickness of the ascending aortic wall was increased with elastic fiber degeneration and collagen deposition, and the number of vasa vasorum in the adventitia was increased compared to that in the control specimen from a heart transplant recipient (Figures 1(c) and 1(d)). Moreover, compared with the control, the phosphorylation levels of Smad2 in the adventitia and ERK1/2 in the media were significantly upregulated (Figures 1(e) and 1(f)), which suggests the activation of the transforming growth factor-*β* (TGF-*β*) signaling pathways in the patient.

### 3.2. Segregation Analysis

**Figure 2:**
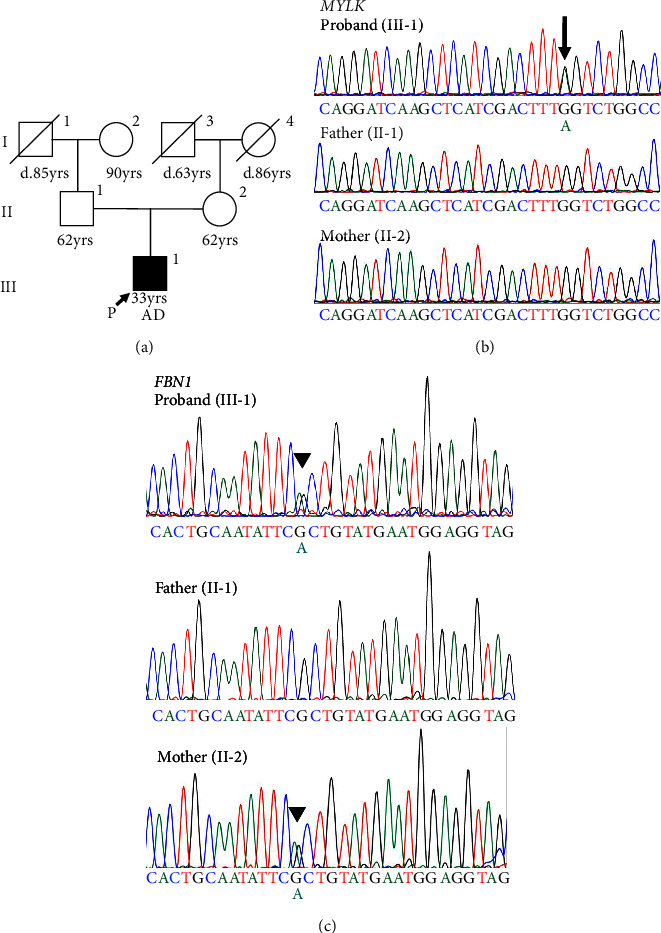
Segregation analysis in this case. (a) Pedigree in this proband (III-1). A black symbol indicates that an individual is affected with thoracic aortic aneurysm and dissection, and “d” indicates age at death. Square, male; circle, female; P and arrow, proband; slash line, died; AD, aortic dissection. (b, c) A heterozygous single-base substitution (c.4819G > A, black arrow) in the *MYLK* gene and (c.365G > A, black arrowheads) in the *FBN1* gene have been identified by sequencing analysis, which shows that the proband alone has the *MYLK* variant (black arrow), whereas the proband and his unaffected mother both carry the *FBN1* variant (black arrowheads).

There was no apparent family history of connective tissue diseases (CTDs) or cardiovascular diseases observed in the patient (Figure 2(a)), as well as no physical examination findings suggestive of CTDs, such as MFS. Due to the aortic dissection at an early age, genetic testing for hereditary TAAD was carried out, and two missense variants of undetermined significance were detected in exon 5 of *FBN1* (c.365G > A, p.[Arg122His]; NM_000138.4) and exon 28 of *MYLK* (c.4819G > A, p.[Gly1607Ser]; NM_053025.3). The novel missense variant of *MYLK* is located in the MLCK kinase domain, which is conserved across species, while the *FBN1* variant has been reported as a variant of uncertain significance (VUS) in the ClinVar database (RCV001189618.2). In silico Combined Annotation Dependent Depletion scores for predicting the pathogenicity of both variants are high (28.6 and 26.3, respectively), as well as their Polymorphism Phenotyping v2 scores (0.977 and 0.991, respectively). We conducted the familial cosegregation analysis using Sanger sequencing to investigate the impact of these variants on arteriopathy in this family. Consequently, the missense variant of *MYLK* was detected only in the proband and not in their parents, and therefore this was considered to be a de novo variant (Figure 2(b)). The patient and his mother carried the missense variant of *FBN1*, whereas the patient's father had neither (Figure 2(c)). The patient's mother was 62 years old, and no enlargement of the sinus of Valsalva was seen on echocardiography (31 mm; aortic root Z-score [15], 0.68). According to the American College of Medical Genetics and Genomics (ACMG) guidelines [16] and the Clinical Genome Resource (ClinGen) recommendations [17], the *MYLK* and *FBN1* variants were classified as likely pathogenic (PM1, PM2_Supporting, PM6_Supporting, PP3, and PP4) and VUS, respectively, in the present case.

## 4. Discussion

A young patient with nonsyndromic TAAD was reported in this study. Gene panel sequencing for TAAD-associated genes identified two candidate causal missense variants in *MYLK* and *FBN1*. *FBN1* is the causative gene for MFS, the most common inherited form of syndromic TAAD, but *FBN1* rare variants and polymorphisms have been also reported to contribute to nonsyndromic TAAD [8, 9]. Conversely, pathogenic variants of *MYLK* cause nonsyndromic TAAD, and the natural history and appropriate treatment strategy remain elusive [10].

Personalized aortic surveillance and clinical management would be supported by validation of pathogenicity of gene variants. In the present case, c.4819G > A in *MYLK* was a novel de novo heterozygous variant, and based on the ACMG guidelines and the ClinGen recommendations, it is classified as likely pathogenic. Although most *MYLK* pathogenic variants are truncating variants (e.g., frameshift and nonsense variants) which resulted in premature truncation and nonsense-mediated decay and are thought to cause disease as a consequence of haploinsufficiency, only a few pathogenic missense variants of *MYLK* have been previously reported, two of which remarkably reduce MLCK kinase activity *in vitro* [10, 11]: c.5275T > C (p.Ser1759Pro) in the calmodulin binding site and c.4471G > T (p.Ala1491Ser) in the kinase domain (ClinVar database accession numbers are RCV000023044.4 and RCV000855690.3, respectively). Although c.4819G > A located in the MLCK kinase domain may also have a loss-of-function effect *in vitro*, further research is required in order to fully understand the variant functional effect, since missense variants were associated with a higher risk of first aortic event (elective aortic aneurysm surgery or acute aortic dissection) than truncating variants [18].

The *in vivo* mechanisms through which *MYLK* pathogenic variants cause aortopathy are completely unknown. Activation of ERK1/2 and Smad2 and enhanced vasa vasorum formation were observed in proband's aortic wall; however, these changes might be influenced by aortic dissection, while the increased vascularity in the aortic media has been reported from patients who had nondissecting thoracic aneurysm and *MYLK* pathogenic variants [10, 19]. It is interesting to note that *MYLK* pathogenic variants can cause aortic dissection with little to no enlargement of the aorta [10]. The ascending aorta was mildly dilated as seen on CT at the first examination (diameter 45 mm) in the present case, and 3 months later, it expanded to 55 mm. Although the exact onset time of aortic dissection was uncertain, his aortic size before dissection might have been much smaller than at dissection, since aortic dilatation occurs rapidly in aortic dissection [20].

c.365G > A in *FBN1* might serve as a modifier for the disease. The penetrance of *FBN1* pathogenic variants is reported to be 100% [21, 22], and c.365G > A has been observed in patient with nonsyndromic TAAD [23]; however, currently, there are not enough data to determine the impact of c.365G > A variant on FBN1 protein function and TAAD pathogenesis, which includes MFS. In the present family, the proband did not have pear-shaped aortic root enlargement typical for MFS, and his mother who has c.365G > A did not show any characteristic features of MFS which includes aortic aneurysm. These results lead us to the conclusion that c.4819G > A in *MYLK*, as opposed to c.365G > A in *FBN1*, had a stronger effect on the development of aortic dissection in the proband.

Because there are few reports on *MYLK* pathogenic variants, the best course of treatment and indications for prophylactic aortic surgery have not, to date, been convincingly established. Although genetic testing has grown more accessible, faster, and affordable, uncertainty in variant interpretation can result in incorrect diagnosis and prevent appropriate therapy and genetic counseling, especially when multiple candidate variants are identified as it happened this time. Better treatment options for rare diseases like nonsyndromic hereditary TAAD could be established by accumulating data on the pathogenicity of rare variants by performing a thorough pedigree analysis.

In conclusion, we reported a Japanese case of nonsyndromic TAAD with a novel likely pathogenic variant of *MYLK* (c.4819G > A), simultaneously carrying *FBN1* c.365G > A VUS. When genetic testing identifies multiple candidate disease-causal variants, segregation analysis may help detect high-risk patients in which careful treatment strategies are needed.

## Data Availability

The data that support the findings of this study are available from the corresponding author upon reasonable request.
